# A Novel Nonsense Pathogenic *TTN* Variant Identified in a Patient with Severe Dilated Cardiomyopathy

**DOI:** 10.3390/cimb45030157

**Published:** 2023-03-15

**Authors:** Caterina Micolonghi, Marco Fabiani, Erika Pagannone, Camilla Savio, Marta Ricci, Silvia Caroselli, Vittoria Gambioli, Beatrice Musumeci, Aldo Germani, Giacomo Tini, Camillo Autore, Antonio Pizzuti, Vincenzo Visco, Speranza Rubattu, Simona Petrucci, Maria Piane

**Affiliations:** 1 Department of Experimental Medicine, Faculty of Medicine and Dentistry, Sapienza University of Rome, 00161 Rome, Italy; 2 Human Genetics, ALTAMEDICA, 00198 Rome, Italy; 3 Department of Clinical and Molecular Medicine, Faculty of Medicine and Psychology, Sapienza University of Rome, 00189 Rome, Italy; 4 S. Andrea University Hospital, 00189 Rome, Italy; 5 Reproductive Genetics, Juno Genetics, 00188 Rome, Italy; 6 San Raffaele Cassino, 03043 Frosinone, Italy; 7 Medical Genetics Unit, IRCCS Mendel Casa Sollievo della Sofferenza, 71013 San Giovanni Rotondo, Italy; 8 IRCCS, Neuromed, 86077 Pozzilli, Italy

**Keywords:** dilated cardiomyopathy, genetic, TTN, nonsense pathogenic variant, M-band region, sudden cardiac death

## Abstract

Both genetic and environmental factors contribute to the development of dilated cardiomyopathy. Among the genes involved, *TTN* mutations, including truncated variants, explain 25% of DCM cases. We performed genetic counseling and analysis on a 57-year-old woman diagnosed with severe DCM and presenting relevant acquired risk factors for DCM (hypertension, diabetes, smoking habit, and/or previous alcohol and cocaine abuse) and with a family history of both DCM and sudden cardiac death. The left ventricular systolic function, as assessed by standard echocardiography, was 20%. The genetic analysis performed using TruSight Cardio panel, including 174 genes related to cardiac genetic diseases, revealed a novel nonsense *TTN* variant (*TTN:*c.103591A > T, p.Lys34531*), falling within the M-band region of the titin protein. This region is known for its important role in maintaining the structure of the sarcomere and in promoting sarcomerogenesis. The identified variant was classified as likely pathogenic based on ACMG criteria. The current results support the need of genetic analysis in the presence of a family history, even when relevant acquired risk factors for DCM may have contributed to the severity of the disease.

## 1. Introduction

Dilated cardiomyopathy (DCM) is a cardiac disease characterized by left ventricular dilatation and systolic dysfunction. Affected patients are typically evaluated for ventricular enlargement using echocardiography. Systolic dysfunction, which is brought on by a decrease of the myocardial force of contraction, is characterized by a reduced left ventricular ejection fraction (LVEF) [1]. Patients with DCM may live years without any symptoms. On the other hand, heart failure, arrhythmias, and thromboembolic disease are the most common manifestations in symptomatic patients [1]. DCM can result from both acquired myocardial insults (such as ischemic injury, myocarditis, alcohol use, toxins, pregnancy, or metabolic diseases) and genetic causes (30–48% of cases) [2]. In young people, DCM can be included in the context of syndromic DCM, different from the non-syndromic DCM. Syndromic DCM is characterized by other systemic involvement, such as inherited metabolic and neuromuscular diseases, chromosome abnormalities, and genetic syndromes, such as Duchenne/Becker muscular dystrophy, hemochromatosis, and mitochondrial DCM [3]. The genetic causes of non-syndromic DCM include damaging variants affecting a wide variety of genes encoding proteins related to the cytoskeleton, sarcolemma, and nuclear membrane, which cause a deficit in generating and transmitting myocyte contraction and signaling. The detection of two disease-causing mutations, even if rare, has been reported in dilatative cardiomyopathy [4]. According to the Clinical Genome Resource (ClinGen), 11 genes were classified as having definitive evidence for DCM: *TTN* (OMIM*188840), *LMNA* (OMIM*150330), *MYH7* (OMIM*160760), *FLNC* (OMIM*102565), *BAG3* (OMIM*603883), *TNNT2* (OMIM*191045), *RBM20* (OMIM*613171), *SCN5A* (OMIM*600163), *DES* (OMIM*125660), *PLN* (OMIM*172405) and *TNNC1* (OMIM*191040) [5,6,7,8,9,10,11,12,13,14,15,16]. *TTN* is one of the largest human genes, with 363 exons. It encodes titin, a huge muscle protein that covers half of the sarcomere from the Z-line to the M-line, and it is found in both cardiac and skeletal muscles. The titin protein creates structural, mechanical, and regulatory support, with an important role in passive and active skeletal muscle contractility [17]. Titin is essential for the development of muscles, for the transmission of force at the Z-line, and for maintaining resting tension in the I-band region [18]. Since titin is a very large protein, missense heterozygous variants of the encoding gene do not cause damaging changes in protein structure, and only null variants are causative of autosomal dominant DCM (DCM1G; OMIM#604145). *TTN* has the potential to generate over one million splice variants [19]. Currently, the National Center for Biotechnology Information (NCBI) database describes only seven human TTN isoforms. The meta transcript is the longest canonical isoform of 3960 kDa, although it has never been experimentally confirmed. In the adult myocardium, the two major representative isoforms are N2BA and N2B transcripts, which include 313 and 191 exons, respectively [17,20]. The sarcomere is covered by these two major isoforms from the Z-disk to the M-band.

In this report we describe a novel truncating *TTN* heterozygous variant, NM_001267550.2:c.103591A > T (p.Lys34531*), which was identified in a proband with severe DCM and positive family history for DCM.

## 2. Materials and Methods

The proband was referred for genetic counseling and analysis to the Medical Genetic Unit, Sant’Andrea Hospital, Rome. Genomic DNA was extracted from the peripheral blood using the DNeasy Blood & TissueKit and QIAamp DNA Blood Mini Kit (Qiagen, Hilden, Germany), according to the manufacturer’s instructions. Genetic analysis was performed using a TruSight Cardio panel (Illumina, San Diego, CA, USA) according to the manufacturer’s instructions. The panel includes 174 genes related to cardiac diseases, and all core genes associated with DCM. The targeted regions underwent paired-end sequencing (2 × 150 bp cycles) on the MiniSeq platforms. For variant identification, we used a developed pipeline according to the Genome Analysis Toolkit (GATK) best practices for germline variant identification [21,22]. Raw data from next generation sequencing (NGS) was aligned to the human genome reference GRCh37/hg19. BaseSpace Variant Interpreters (Illumina, San Diego) were used for variant prioritization and annotation. Variants with read depth < 10×, quality < 200, synonymous and intronic variants in non-splice regions and minor allele frequency (MAF) higher than 0.01 were removed for further analysis. The identified variants were classified according to the American College of Medical Genetics and Genomics criteria [23]. New identified variants were analyzed using different prediction software (SIFT, Polyphen2 and REVEL) and classified according to both their agreement and the presence of studies in the literature and public databases (ClinVar, HGMD, OMIM, dbSNPs). The identified variant in *TTN* was validated using Sanger sequencing with a set of primers (Forward: 5′-GGAACTGAAAGTGTACCACTG-3′, Reverse: 5′-GGACAGTTTAGGAATACGCCA-3′). The PCR was performed in a 50-μL reaction containing a final concentration of 1× PCR Buffer (Applied Biosystems, Foster City, CA, USA), 50 μmol/L each of dNTP, MgCl2 1.5 mM, 1.25 AmpliTaq Gold (Applied Biosystems), and 0.2 μmol/L each forward and reverse primers. The reaction mixture was subjected to a temperature of 95 °C for 5 min; followed by 35 cycles of 95 °C for 15 s, 60 °C for 15 s, and 72 °C for 1 min; followed by 72 °C for 7 min. The cycle sequencing was performed using the BigDye version 3.1 terminator cycle-sequencing kit, according to the manufacturer’s instructions (Applied Biosystems). The cycle-sequencing conditions were 95 °C for 30 s; followed by 35 cycles of 95 °C × 15 s, 50 °C × 15 s, and 60 °C × 4 min. The products were analyzed using a SeqStudio Genetic Analyzer (Applied Biosystems). The PCR products were also identified on 2% agarose gel electrophoresis and 0.5× TAE gel (40 mM Tris–HCl, 20 mM acetic acid, 1 mM EDTA, pH 8.0), containing GelRed^®^ (1 μg/mL) for DNA staining and visualization. For the calculation of the DNA fragment, a molecular-weight DNA size marker (1 Kb Plus DNA Ladder, Invitrogen^®^, Carlsbad, CA, USA) was included in each gel run. The study was conducted in accordance with the Declaration of Helsinki, and the protocol was approved by the Ethics Committee of S. Andrea Hospital (approval identification number: 42 of 28 September 2007).

## 3. Case Presentation

**Figure 1:**
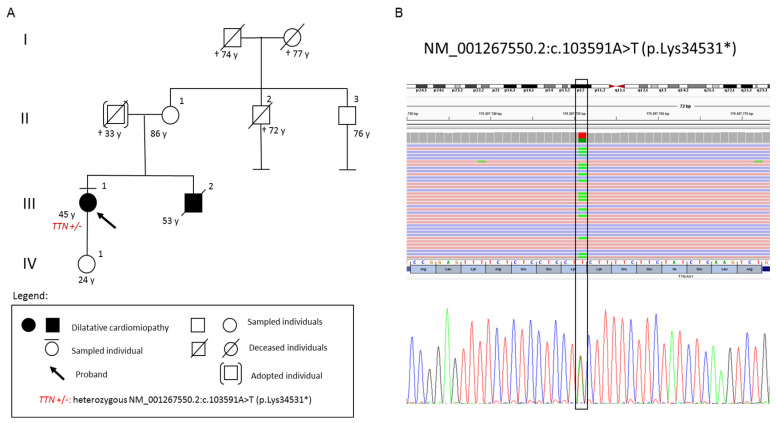
(**A**) Pedigree of the family showing the phenotypes of the affected relatives and the genotype of the proband. Male is represented by a square; female is represented by a circle. (**B**) Multi-gene panel analysis. NGS and Sanger sequencing of the proband showing the c.103591A > T (p.Lys34531*) in *TTN* on genomic DNA. The identified pathogenic variant in *TTN* is visualized by Integrative Genome Viewer (IGV) software. Ref Seq (Reference sequencing) used for variant annotation: NM_001267550.2.

The proband was a 57-year-old woman: an active smoker, with a history of cocaine abuse, affected by arterial hypertension and diabetes. The patient was diagnosed with DCM in our center for “Diagnosis and Treatment of Cardiomyopathies and Hereditary Arrhythmogenic Diseases”, Sant’Andrea Hospital, Rome. She referred a family history of DCM (brother with a diagnosis of DCM deceased at the age of 53 from refractory heart failure), and of sudden death (father died suddenly at the age of 33) (Figure 1). Cardiological examinations were referred within the normal limits until 2017.

**Figure 2:**
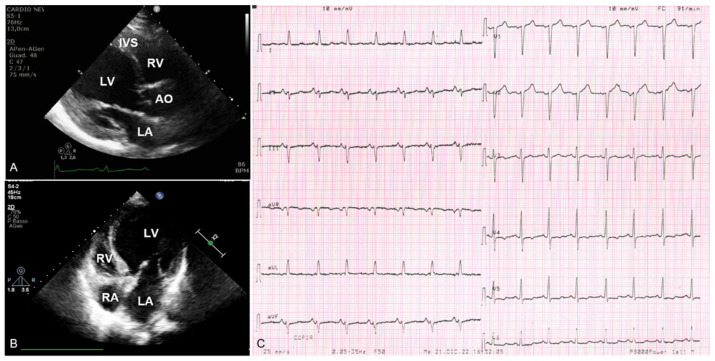
Echocardiographic and electrocardiographic images of the proband (III:1). (**A**) Two-dimensional transthoracic echocardiogram diastolic frames of parasternal long-axis view and (**B**) 4-chamber view showing dilated and globular-shaped left ventricle, with septal thinning. (**C**) Electrocardiogram showing left anterior hemiblock and slight ST segment depression from V4 to V6. LV—left ventricle; RV—right ventricle; LA—left atrium; RA—right atrium; AO—aorta; IVS—interventricular septum.

In November 2022, due to the sudden onset of shortness of breath, heartburn, and desaturation (SpO2 75%), she was escorted to the emergency room of our hospital. Her blood pressure was 140/65 mmHg, and heart rate was 84 bpm. An arterial blood gas analysis showed type 1 respiratory failure (pH 7.41, pO2 49 mmHg, pCO2 37 mmHg, SpO2 83.9%). A chest X-ray revealed a pulmonary oedema. A first-line laboratory work-up showed neutrophilic leukocytosis (WBC 12,400 u/L, neutrophils 79.8%); hyperglycemia (328 mg/dL); slightly increased high sensitivity troponins I (25 pg/mL); and increased B-type natriuretic peptide (BNP) (700 pg/mL) and D-Dimer (689 pg/mL) levels. The electrocardiogram (EKG) showed sinus tachycardia; left anterior hemiblock; and repolarization abnormalities from V4 to V6, which included slight ST segment depression suggestive of subendocardial ischemia or structural heart disease (Figure 2C). The echocardiography revealed an enlarged left ventricular end diastolic diameter (LVEDD 58 mm) and diffuse hypokinesia with severely reduced systolic function (LVEF 25%); right sections were within the normal limits; dilated (20 mm) and no collapsible inferior vena cava. Oxygen therapy and intravenous furosemide and nitrates were administered, with subsequent clinical benefit. The proband was then admitted to the cardiology unit, hemodynamically stable and asymptomatic at rest. The EKG was unchanged. The echocardiogram confirmed a dilated and spherical left ventricle, septal thinning, and severely reduced ejection fraction (LVEF 20%). Given the multiple risk factors and the clinical onset, to rule out coronary artery disease as the cause of the left ventricular dysfunction, the proband underwent coronary angiography that showed diffusely atheromatic coronary arteries without any hemodynamically relevant obstruction. A cardiac MRI was performed with the following report: “enlarged left ventricle, end diastolic volume (EDV): 263 mL, EDV/body surface area (BSA) 162 mL/m^2^, BSA-indexed LVEDD: 36.02 (3.6 cm/m^2^), apical thinning, mass augmentation (mass/BSA: 52 mL/m^2^), diffuse septal hypokinesia and, to a lesser extent, hypokinesia of the basal and medial regions of the anterior and inferior walls, severe depression of ejection fraction (LVEF 22%); normal extracellular volume values.; no late enhancement of contrast areas”. Based on the imaging, the proband received the diagnosis of DCM. The EKG monitoring did not show any arrhythmic episode. Heart-failure-optimized medical therapy was prescribed, and the patient was finally dismissed. At the subsequent clinical re-evaluation within one month, she was clinically stable, her New York Heart Association class (NYHA) was I–II, with referred full adherence to the prescribed medical therapy. The EKG showed a lower heart rate but it was otherwise unchanged. The echocardiogram showed a globular shaped, dilated left ventricle (LVEDD 58 mm), with septal a-dyskinesia and hypokinesia of the remaining walls; severe systolic dysfunction (LVEF 30%); a left atrium diameter at the upper limits (39 mm), but normal right sections; first degree diastolic dysfunction, with mild mitral and tricuspidal regurgitation; and sclerotic aortic root and aortic valve, with minimal aortic regurgitation. (Figure 2A,B).

Medical therapy was further optimized. A 24 h Holter EKG was recommended and a further follow-up was scheduled in the outpatient clinic to evaluate the indication for a defibrillator implantation for primary prevention of sudden death. To investigate the genetic causes of the disease in the proband, genetic tests were performed after obtaining ethical approval and written informed consent. The genetic analysis identified a novel truncating heterozygous variant NM_001267550.2:c.103591A > T (p.Lys34531*) in *TTN*. The frequency of this variant in the general population is unknown and absent in all investigated databases (dbSNP, HGMD, Clinvar, Varsome, OMIM, FRANKLIN, and LOVD). The PhyloP100way score of the c.103591A > T (p.Lys34531*) identified variant suggests the important evolutionary conservation of this site. According to the American College of Medical Genetics and Genomics (ACMG) criteria (PVS1, PM2) the variant was classified as likely pathogenic [24]. The segregation study of the identified variant in the proband could not be performed since the affected brother (III:2) had recently died, and neither the asymptomatic mother (II:1) or daughter (IV:1) were available for blood sampling at the time of the study.

## 4. Discussion and Conclusions

DCM is a major cause of heart failure and has a genetic basis in 38–40% of cases. Titin truncating variants (*TTN*tv) lead to several diseases due to alterations of the protein’s biological function [25]. *TTN*tv explain 25% of all genetic causes of DCM [26]. Dissecting the pathological phenotype derived from the different mutations is challenging. The introduction of novel sequencing technologies, such as NGS, has allowed researchers to identify new *TTN* variants and to study the relationship between genotype and clinical phenotype [27].

*TTN*tv also occur in the general population (1%), making it difficult to determine their pathogenic role in DCM. According to Roberts and colleagues [28], the pathogenicity of *TTN*tv is closely related to (i) the isoform in which it is included, as those located in the minor *TTN* isoforms are more frequent in healthy populations; (ii) the expression of the exon in which it is located, as those in the constitutively expressed ones are strongly linked to DCM, and (iii) the variant position, with the more C-terminal variants being more pathogenic [28,29,30]. The exon 359 is included in all *TTN* transcripts (PSI 100%) and is asymmetric, since its length is not a multiple of three and therefore its removal will alter the reading frame.

Differences in phenotypes and clinical outcomes in patients with unique pathogenic variants in *TTN* are still unclear. Some studies demonstrated that baseline clinical phenotypes and the age of onset of LV systolic dysfunction are not statistically different from one another when the truncating variant is located in various *TTN* bands or positions [31]. In fact, *TTN*tv-related DCM seems to be similar or less severe than other types of DCM. Indeed, many authors assess a milder phenotype and a better outcome of *TTN*tv carriers compared to idiopathic or other forms of hereditary DCM. Moreover, many *TTN*tv patients have an improvement of their systolic disfunction and favorable outcomes in reverse remodeling, after therapy [32,33,34]. On the other hand, other investigators suggested a worse cardiac phenotype when the truncating variant falls in either of the two main isoforms of *TTN* and/or resides toward the carboxy-terminus, such cases being responsible for DCM with severely impaired LV function and life-threatening ventricular arrhythmias [28]. The female gender may influence the effect of TTNtv mutation [35].

In this report, we describe a novel nonsense variant NM_001267550.2:c.103591A > T in exon 359 of *TTN* occurring in the M-band region of the titin protein in a patient with severe DCM, acquired risk factors for DCM, and positive family history of DCM.

The M-band region is a highly sophisticated region including Ig-like domains, fibronectin type-III, and a protein kinase domain, which helps to maintain the regular lattice of thick filaments. Titin joins the thick filament in the A-band, integrates into the M-band via myomesin, and then forms a continuous filament with titin in the neighboring sarcomere M-band [36,37]. The protein kinase domain of the M-band is involved in strain-sensitive signaling and is implicated in the sarcomerogenesis as well as in the cardiac remodeling in DCM [38,39]. It is known that heterozygous *TTN* variants in the M-band can be the cause of DCM. Indeed, other variants in this region were described in DCM patients in different cohorts [28,40]. Despite its small size (only 6.5% of the gene), the M-band region appears to be a mutational hotspot because a high number of variants falling within the M-band were classified as pathogenic in the literature. However, this information could be biased by several previous screenings performed in this region in patients with skeletal muscle diseases [17]. Numerous *TTN* variants were identified as pathogenic, but their effects on transcripts and protein function, particularly regarding either the loss or the gain of titin function, have not yet been fully understood. Non-truncating (often missense) variations offer challenging interpretations because of their presence in crucial functional regions (such the titin kinase domain) and in contact domains with several other interacting proteins involved in cardiomyopathy, such as myosin-binding protein C (MyBP-C) [41]. Clinical and molecular evidence showed that *TTN*tv contributed to disease with combined dominant-negative and haploinsufficiency of the full-length titin protein in DCM patients [42,43]. In these patients, the variant results in a truncated form of the titin protein, which leads to sarcomeric instability and the development of DCM [40]. Variants falling near the one identified in our patient (*TTN*:c.103591A > T), located around the carboxy-terminus in high PSI exons, compared to proximal truncations in exons with low PSI, were linked to worse ventricular contractile function, decreased indexed stroke volume, and decreased EF of both left and right ventricles, resulting in reduced LV function and potentially fatal ventricular arrhythmias [28,40]. The severe phenotype of our patient could also be the consequence of environmental risk factors. In fact, untreated diabetes, hypertension, smoking habit, and alcohol and cocaine abuse are known factors responsible for acquired DCM [44].

The potential utility of genetic analysis is growing in all cases of cardiomyopathy with known risk factors. Patients with a genetic predisposition may remain phenotypically silent until an environmental trigger occurs. The *TTN*tv clinical phenotype is also particularly susceptible to environmental factors, which can favor the onset of the disease [45,46,47]. At first sight, the present case could be classified as an acquired form of DCM. However, the family history was a crucial indication to undertake genetic testing, leading to the identification of the genetic cause of the disease and allowing the patient’s daughter to carry out the test on her own for preventive and surveillance purposes. Therefore, genetic testing should always be considered in the diagnostic approach of DCM in the presence of a family history, even when important risk factors (drugs, alcohol abuse, etc.) are also present, similarly to what has been considered in other cardiac diseases [48].

In conclusion, our case report supports the pathogenicity of a novel *TTN* variant. In the described case, the effect of the c.103591A > T variant can contribute to the DCM phenotype in a variable manner, along with other genetic and environmental factors. Genetic counseling and comprehensive genetic testing can help determine the specific implications of this mutation in each affected individual. This article provides new insights into the genetic basis of DCM and the role of the *TTN* gene in the development of this disease. These findings may have important implications for the diagnosis, management, and treatment of DCM in patients with *TTN* truncations. Further research is needed to fully understand the impact of *TTN* truncations on the development and progression of DCM.

## Data Availability

The authors are available to share results upon request.
